# Honey bees save energy in honey processing by dehydrating nectar before returning to the nest

**DOI:** 10.1038/s41598-022-20626-5

**Published:** 2022-09-28

**Authors:** Susan W. Nicolson, Hannelie Human, Christian W. W. Pirk

**Affiliations:** Department of Zoology and Entomology, University of Pretoria, Pretoria, 0002 South Africa

**Keywords:** Ecology, Physiology

## Abstract

Honey bees process nectar into honey by active evaporation on the tongue and passive evaporation involving nest ventilation and fanning behaviour, as well as enzymatic action. The elimination of excess water from nectar carries considerable energetic costs. The concentration of the nectar load is assumed to remain constant during transport. However, some of this water elimination may occur before foragers return to the nest and pass their nectar loads to receiver bees. In honey bees captured while foraging in *Macadamia* orchards, we show that the nectar in their crops has approximately twice the sugar concentration of the fresh nectar in flowers. This was true for four *Macadamia* cultivars, with up to 75% of the initial water content being removed. There is a further concentration increase in the crops of returning bees captured at the hive entrance. The only possible route of water elimination from the crop is via evaporation from the mouthparts. We calculate the savings in honey processing costs to be on average 35 times more than the reduction in flight costs due to reduced body mass. Pre-concentration of nectar in foraging honey bees may be widespread, and of crucial importance for honey storage.

## Introduction

The conversion of nectar into honey involves the removal of large amounts of water. Nectar collected at concentrations ranging from 20 to 60% w/w must be concentrated to more than 80% w/w for storage in the nest^1,2^. Returning foragers pass their nectar loads by trophallaxis to receiver bees. Individual bees first actively evaporate the nectar by repeated regurgitation and re-ingestion of nectar droplets, also known as ‘tongue lashing behaviour’, which occurs for extended periods (described in detail by Park^3^). This is followed by passive evaporation, aided by fanning behaviour of large numbers of bees, from droplets placed in cells and relocated between partially filled cells before final storage^4,5^. Both active and passive evaporation mechanisms are aided by the high surface area to volume ratio of small droplets. Apart from the physical process of evaporation, the conversion of nectar into honey involves sucrose hydrolysis, with enzymes being added to nectar in the crop during collection and processing^1,6^.

Honey ripening is assumed to occur only after returning foragers transfer their nectar loads to receiver bees. Early studies showed that the concentration of nectar does not increase while honey bee foragers transport it from flowers to the hive; rather there may be slight dilution due to the addition of saliva^7^. However, elimination of excess water can begin during transport to the hive. *Aloe greatheadii* var. *davyana* is an important winter bee plant in South Africa and its relatively dilute nectar (20% w/w) contrasts with a sugar concentration almost twice as high in the crop contents of bees captured at flowers (after foraging for at least 20 s) and on return to the hive^8^. The doubling of the concentration suggests that nectar is regurgitated onto the tongue and evaporated during foraging and flight. If the bee crop were not impermeable to water, the steep osmotic gradient between crop contents and haemolymph^9^ would cause dilution of the crop contents: this leaves evaporation from the tongue as the only possible route of water loss^8^.

For honey bees, the pre-concentration of dilute nectar may offer energetic advantages. Partial crop loading may be due to the metabolic cost of load carrying^10,11^, shown by direct measurements in individual honey bees carrying different masses of sugar solution^12^. On the other hand, there may be energetic benefits at the colony level in view of the considerable cost of drying nectar. Mitchell^13^ has recently demonstrated that the dehydration of nectar is a surprisingly energy-intensive process, with 25–60% of the sugar in the nectar being metabolised in the process. The energy required for dehydration depends on the nectar concentration, the distance between nectar source and nest, ambient temperature and the thermal conductance of the nest^13^. This work on the energetics of nectar dehydration calls for further examination of possible pre-concentration of nectar by bees before return to the nest.

The pre-concentration of freshly collected nectar by honey bees may be more widespread. *Macadamia integrifolia* (Proteaceae) is an important nut crop in South Africa, flowering in spring, and pollination by honey bees (*Apis mellifera scutellata*) is important for crop yields (and honey production). Using data on the volume and concentration of the crop contents of bees foraging on *Macadamia* flowers, here we test the following hypotheses: (1) a high proportion of the water in nectar is evaporated before return to the nest; (2) the resulting higher concentration of the crop contents reduces the load to be carried and thus flight costs; and (3) the higher concentration means that less evaporation is needed for conversion to honey, so honey processing is less expensive.

## Results

**Figure 1:**
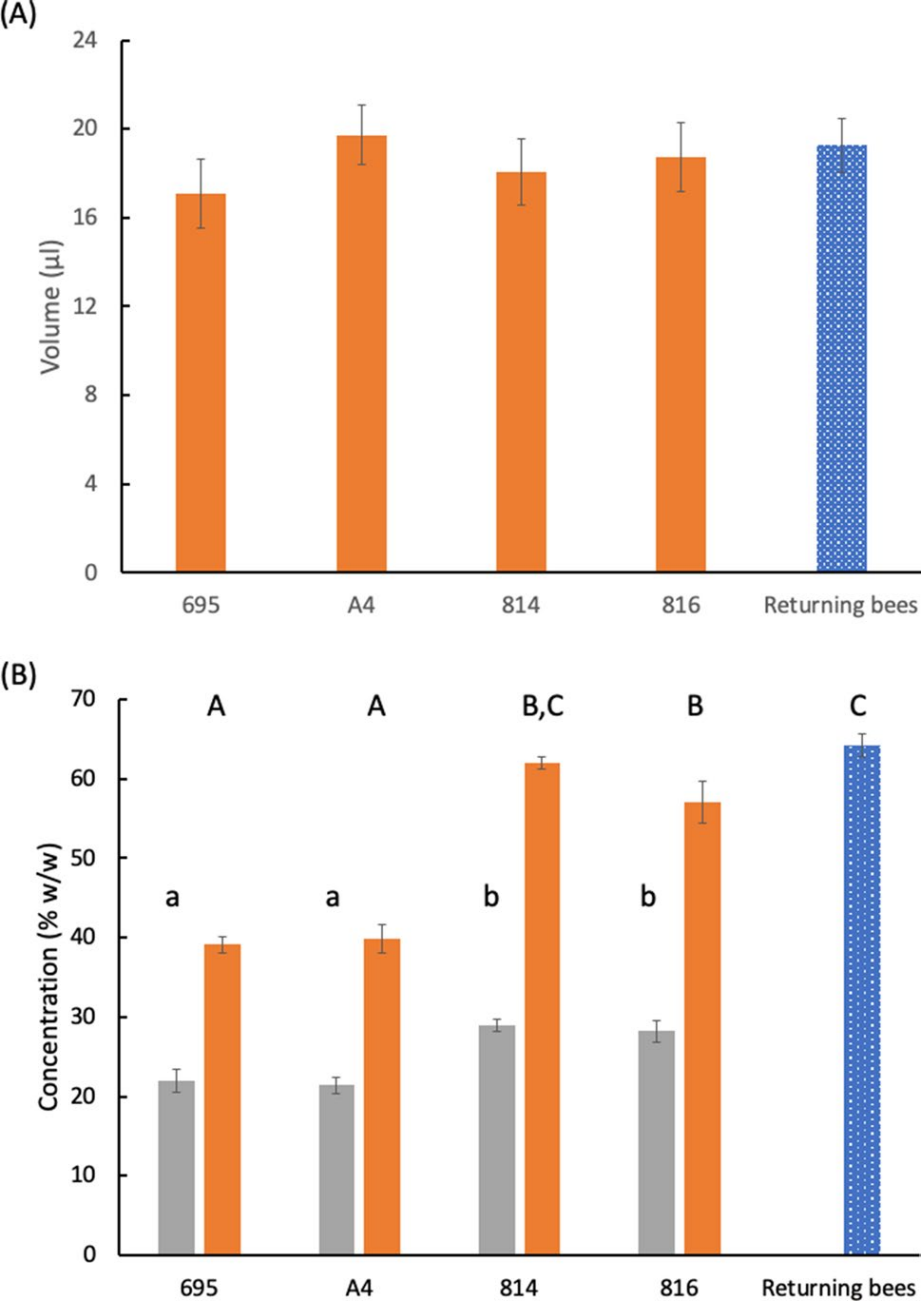
Crop contents of honey bees foraging on four cultivars of *Macadamia integrifolia* and captured on return to the hive. Nectar concentrations of the four cultivars are included. Values are means ± 95% CI (n = 30). (**A**) Volume in µl. (**B**) Concentration in % w/w of crop contents (orange) and nectar of the same cultivar (grey). Different letters indicate significant differences in crop contents; small letters refer to differences in nectar concentrations. Significant differences were determined by overlapping CIs between two groups.

We studied honey bees foraging on four different cultivars in macadamia orchards. Nectar was sampled from flowers of each cultivar, and on the same day foraging honey bees (*Apis mellifera scutellata*) were captured on flowers and their crops removed for measurement of volume and concentration of the contents. Returning bees were also sampled at the hive entrance, but data for their crop contents were pooled for the four cultivars as it was not possible to determine which cultivar the bee had been visiting (Supplementary Table S1). The volume of nectar in crops of bees foraging on the various cultivars and those returning to the hive showed no significant differences (KW-ANOVA H = 5.38, *p* > 0.05, Fig. 1A). The concentration of nectar present in the crops of foragers at the various cultivars displayed significant variation (KW-ANOVA H = 110.57, *p* < 0.001, Fig. 1B). The nectar concentration in flowers was similar between cultivars 695 and A4 but both differed significantly from cultivars 814 and 816 (Fig. 1B). Crop concentration in cultivar 816 was also significantly lower compared to returning foragers (Multiple comparison, z = 3.36, *p* < 0.008, Fig. 1B). The lowest average concentration was recorded in cultivar 695 and the highest concentration in bees returning to the hive (Fig. 1B).

Table 1 presents data on the masses of sugar and water in the crops of bees sampled on the four cultivars, and the mass of water in the crops assuming constant sugar mass but the original nectar concentration. The total mass of sugar present in bee crops differed significantly (KW-ANOVA H = 79.63, *p* < 0.0001): cultivars 695 and A4 were not significantly different from each other but were significantly lower compared to the other three groups. The mass of sugar was highest in cultivar 814 and honey bees returning to the hive and lowest in cultivar 695 (Supplementary Table S2). The mass of the crop contents, obtained from the volume and density of the sugar solution^14^, was significantly different among the groups (KW-ANOVA H = 14.37, *p* < 0.007) but the pairwise comparison showed only that returning bees had a significantly higher mass of the crop contents than cultivar 695 (Multiple comparison, z = 3.66, *p* < 0.003). The mass of water in the crops showed a similar picture to total sugar mass (KW-ANOVA H = 57.19, *p* < 0.0001): cultivars 814 and 816 and returning bees were similar but significantly lower than A4, and cultivar 695 was similar to A4 and 816 but significantly higher than 814 and returning bees (Supplementary Table S2). Without evaporation from the crop contents during foraging, the mass of the crop contents (KW-ANOVA H = 57.1, *p* < 0.0001) and the mass of water in the crop (KW-ANOVA H = 50.94, *p* < 0.0001) were significantly different among the groups. Returning bees had a significantly higher mass of crop contents compared to all other groups and cultivar 695 was significantly lower compared to all but A4 (Supplementary Table S2). Regarding the mass of water in the crop, returning bees again had significantly higher mass than any other group and cultivar 814 was significantly different from 695 (Supplementary Table S2). The highest amount of water evaporated was estimated for returning bees at the hive entrance followed by cultivars 814 and 816, then cultivars A4 and 695 with the lowest amount (KW-ANOVA H = 76.76, *p* < 0.0001). Returning bees were significantly higher compared to all groups, and cultivar 814 differed significantly from 695 and A4 but not 816, whereas 816 was only significantly higher than 695 (Supplementary Table S2). Table 1 shows that over 70% of the initial water content was removed by honey bees foraging on the two cultivars (814 and 816) with higher nectar concentrations (Fig. 1B).

**Table 1:** Mass of sugar, water and crop contents in honey bee crops with and without evaporation.

Cultivar	695	A4	814	816	Returning
Mass of sugar	7.9 a	9.3 a	14.7 b	13.6 b	16.3 b
CI	0.81	0.78	1.27	0.15	1.12
Mass of crop contents	20.1 a	23.3 a,b	23.6 a,b	23.9 a,b	25.4 b
CI	1.84	1.57	1.95	2.07	1.62
Mass of water	12.2 a,c	14.0 a	8.9 b	10.3 b,c	9.0 b
CI	1.07	0.99	0.72	1.91	0.67
Mass of crop contents (no evap)	35.9 a	43.4 a,c	50.7 b,c	48.7 b,c	64.9 d
CI	3.69	3.63	4.38	5.28	4.47
Mass of water (no evap)	28.0 a	34.1 a,b	36.0 b	35.0 a,b	48.6 c
CI	2.88	2.85	3.11	3.79	3.34
Mass of water evaporated	15.8 a	20.1 a,c	27.1 b	24.7 b,c	39.6 d
CI	1.81	1.86	2.39	1.88	2.99
% water evaporated	56.5	59.1	75.3	70.7	81.4

Values in mg (mean and 95% confidence interval, n = 30). Different letters within a row indicate significant differences.

**Figure 2:**
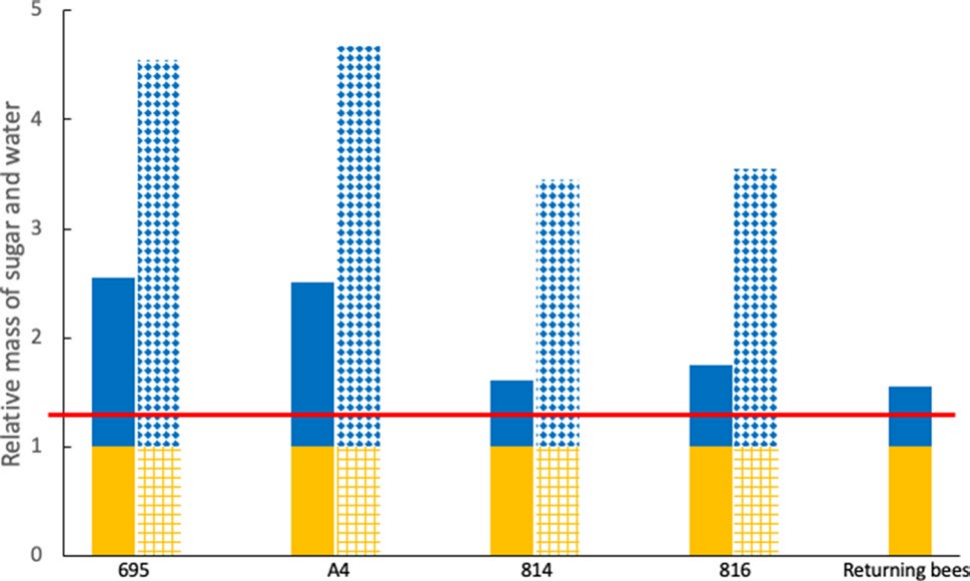
Relative masses of sugar and water in crop contents of honey bees. Masses of sugar (yellow) and water (blue) are shown for bees foraging on four *Macadamia* cultivars, compared with the estimated values if no evaporation occurred. Estimated values (not solid) are based on crop contents remaining at the same concentration as nectar. Foragers returning to the hive are also included. The red horizontal line indicates the composition of honey (80% w/w sugar).

Further evaporation of water apparently occurred during the return flight to the hives. Crop contents of bees captured at the hive entrance had a mean sugar concentration of 64% w/w. Using the mean nectar concentration for the four cultivars (because the data for returning foragers was pooled) we find that an overall 81.5% of the water in the nectar was removed. Figure 2 shows the masses of sugar and water in the crops of foraging bees and those returning to the hive: the mass of sugar for each cultivar remains constant but the mass of water varies dramatically between the measured value and the value estimated assuming evaporation does not occur.

We calculated the savings in flight costs due to reduced crop volumes, and the savings in honey processing costs. The reduction in flight costs due to evaporation of the crop contents was calculated from the metabolic rates of bees carrying full or reduced crop loads (see Methods). Calculation of the energy used to return the nectar load compared to the energy that a worker would need to return the same amount of sugar but without evaporation shows that the bee would invest on average between 13 and 19% more energy depending on the variety (Fig. 2).

**Figure 3:**
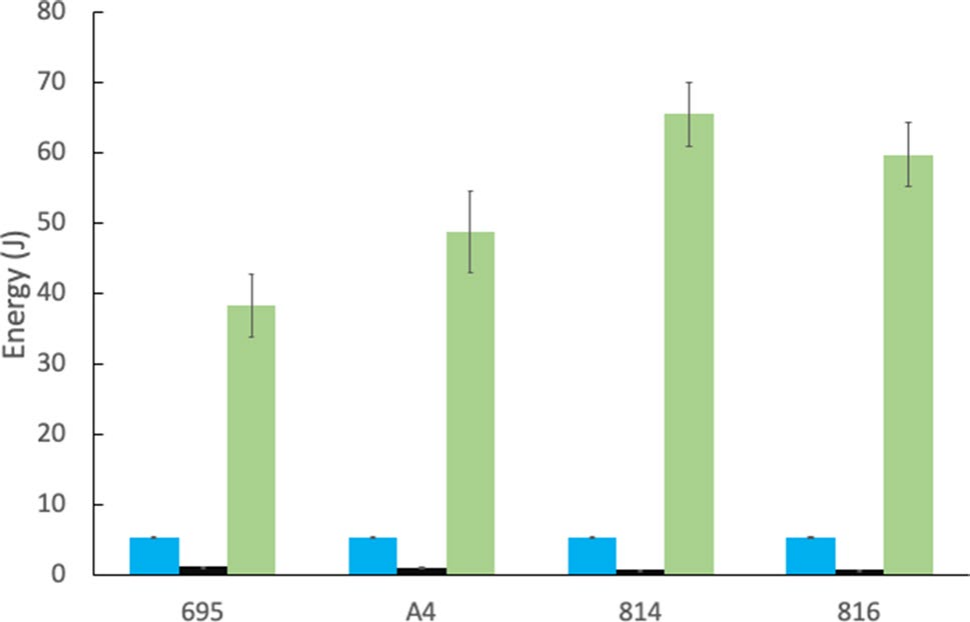
Energy benefits from evaporating nectar during foraging and flight. Average energy (J) needed to transport the reduced crop load back to the hive (blue), energy saved due to reducing body mass by evaporation of crop contents (black) and energy needed to evaporate the excess water in the hive (green). Values are shown for the four cultivars (n = 30 bees per cultivar).

The savings in honey processing costs, calculated using the heat of vaporisation of water, were much greater. Our estimate of the amount of water evaporated ranged on average between 16 and 27 mg, depending on cultivar (Table 1) and this mass of water would cost an additional 35–65 J of energy (Fig. 3) to be evaporated in the nest. The energy needed to evaporate the excess water far exceeded the energy costs of flight, whether the nectar load was full or reduced (Fig. 3).

## Discussion

The nectar desiccation process appears to begin during foraging in honey bees. This contradicts the conventional wisdom that there is no change in nectar concentration between the flower and the nest, but note that Park’s classic work of 90 years ago was done with artificial nectar sources^7^. In all four *Macadamia* cultivars examined, nectar in the crops of honey bees had approximately doubled in concentration, compared to nectar in flowers, while bees were still on the flowers. As in the case of aloes^8^, there was further concentration of the crop contents on the return flight to the hive, so that crop contents of bees captured at the hive entrance had a very high mean sugar concentration of 64% w/w. We suggest that the phenomenon may be widespread because honey bees receive remarkable energetic benefits from concentrating the nectar before unloading in the nest. The reduction in flight costs has previously been considered important^10–12^, but is small compared to the reduction in costs of honey processing.

Some immediate concerns should be addressed here. It is possible that bees are choosing florets with the most concentrated nectar. However, the nectar concentrations within each cultivar showed little variation (Fig. 1B). The differences between the maximum nectar concentration recorded for each cultivar (Supplementary Table S1) and the mean concentration of the forager crop contents were substantial, ranging from 9% w/w (cultivar 695) to 31% (cultivar 814). We can also rule out the effect of the nectar load on departure. Honey bees leave the hive with small amounts of concentrated nectar in their crops, to be used as fuel for flight and glue for sticking pollen grains together^15^. However, this amount of nectar sugar is too small to account for the increase in concentration at the flowers. In the aloe study, we recorded departing crop loads averaging 1.1 μl in volume with a concentration of 67.7% (w/w) sugar^8^. Nectar varies in sugar composition, and the nectar of *Macadamia integrifolia* contains only glucose and fructose^16^. Refractometer readings and our calculations on sugar/water proportions are based on sugar masses and thus unaffected by sugar composition^17^. Moreover, sucrose-rich nectars are converted to hexose solutions by enzymatic action in the crop^1,6^.

The energetic cost of evaporating nectar to make honey has been overlooked in considering the energy efficiency of honey production^18^. The modelling study of Mitchell^13^ is an important contribution which demonstrates that the process of evaporation uses at least 25% and often over 50% of the energy in the nectar brought into the nest. This is a consequence of the high heat of vaporisation of water. A key factor is the thermal energy efficiency, which depends on nest conductance, the difference between ambient and nest temperatures, and the nectar concentration. This work on the energetics of nectar dehydration calls for revisiting any possible pre-concentration of nectar by bees before return to the nest, and it is surprising that few measurements are available on the concentrations of bee crop contents. For bees on macadamias, we estimate that 57–75% of the water in the nectar (depending on cultivar) was evaporated while bees were still on the flowers, and an average of 81% on arrival at the hive entrance. During a strong nectar flow this will represent a huge energy saving at colony level.

Honey bee nest humidity depends on the amount and concentration of nectar being concentrated into honey^19^. During nectar flows, most of the water vapour in the nest is a direct consequence of nectar dehydration; this includes metabolic water production and water in the nectar consumed to fuel the evaporation^19^. Detailed recordings of humidity in *A. m. scutellata* hives show that absolute humidity is lower in the nectar storage zone than in the brood zone, but higher than in ambient air^20,21^. These values are in agreement with the conflicting requirements of high humidity for brood development and low humidity for concentrating nectar^22^. Importantly, because absolute humidity is higher in the nectar storage zone than in ambient air, it is more energy efficient for worker bees to evaporate nectar outside than in the nest. The improvement in efficiency will vary with climate and whether colonies are in man-made hives or in more humid tree nests^19,21^.

Other solitary and social bees are known to eliminate water from relatively dilute nectar. Portman et al.^23^ provide evidence that nectar concentrating behaviour is widespread in bees (51 genera in six families), and discuss various potential reasons for this behaviour. These include: preparation of nectar for long-term storage, improving the consistency of larval provisions, improved flight efficiency, more effective binding of pollen grains for transport, and evaporative cooling. In general, a film or droplet of regurgitated crop contents is manipulated on the mouthparts, giving the maximum surface area for evaporation: this is easier to see in stationary bees, such as *Allodapula* or *Euglossa*^24,25^. Video recordings have also shown nectar concentrating behaviour during flight in one species (*Osmia lignaria*) and during pollen collection^23^. We have not been able to observe the process directly in bees foraging on either aloes or macadamias. This is partly because *A. m. scutellata* foragers become very active and aggressive on rich floral resources. However, the manipulation of nectar or water on the tongue of honey bees has been recorded in a thermoregulatory context in observation hives or under laboratory conditions^26–29^. The resulting evaporative cooling was associated with high ambient temperatures. Among honey bees foraging in the Sonoran Desert, a higher proportion returned with droplets of crop contents on the tongue as the ambient temperature increased to 40 °C, and bees with extruded fluid had lower head and thorax temperatures than those without^28^. The thermoregulatory implications of evaporating nectar before return to the hive are unknown: the associated evaporative heat loss will depend on vapour pressure deficits during foraging and may or may not be an advantage to individual foragers^24,29^.

Our findings cast some doubt on crop volumes recorded in the literature. We measured consistently low crop volumes of 17–19 µl in bees on all four cultivars, as well as in returning bees captured at the hive entrance. This is comparable to bees on the aloes where mean crop volumes were 10–20 µl in bees at flowers and returning foragers^8^. It is important to note that 20 µl of concentrated nectar is equivalent to twice the volume of floral nectar. Field records of honey bee crop volumes much lower than capacity are common^30,31^ but are misleading if the crop contains nectar that has been concentrated from a higher volume. Honey bees can carry 60–70 µl in their crops, and higher crop volumes tend to be associated with high volumes of relatively concentrated artificial nectars provided in easily accessible feeders^32–34^. We have also measured crop volumes exceeding 40 µl in *A. m. scutellata* offered dilute sucrose solutions in feeders near the hive^35^. When nectar is difficult to collect from flowers, researchers have sometimes estimated its concentration by catching foraging bees and extracting their crop contents, assuming that there is no change in concentration in the crop^36–38^. Our results for honey bees foraging on aloes and macadamias suggest that this method may greatly overestimate nectar concentrations. There are other alternatives for collection of small volumes, such as the use of filter paper wicks or the rinsing method^39^.

Clearly, there is a need for systematic collection of data comparing nectar concentrations of other plant species with the crop contents of honey bees foraging on them. In addition, future studies utilising artificial feeders would enable the concentration of the collected nectar to be known exactly. Aloes and macadamias have high and low nectar volumes in individual flowers respectively, but for both species nectar concentrations were relatively low. On macadamia flowers, with nectar of 20–30% w/w at the time of sampling, returning foragers had crop contents with a mean sugar concentration of 65%. More water was evaporated (> 70% of the initial amount) from the two cultivars (814 and 816) with higher nectar concentrations: this may be related to vapour pressure deficits during the morning of sampling (see Supplementary Table S1). Weather conditions should be recorded continuously during future measurements of nectar *vs* crop concentrations. Bees foraging on macadamias returned to the hive with higher crop concentrations than those foraging on aloes (38–40%, nectar initially ~ 20%^8^). On *Macadamia* racemes, collecting nectar from many small flowers may provide more time for evaporation from the mouthparts.

At the colony level, the process of offloading nectar is an important part of foraging^40^. Honey bees that pre-concentrate nectar during foraging will deliver very different concentrations from those in nectar. This has implications for information exchange: when higher sugar concentrations are transferred by trophallaxis to nestmates, they will perceive the nectar source as having a higher concentration. However, this effect should be the same for most nectar sources, and we suggest that the colony could benefit from a positive feedback loop. If long foraging distances allow for more evaporation to take place, it might become energetically beneficial to explore more diluted but distant nectar sources. When the bee forages far from the nest, the nectar evaporating benefit for the colony is much greater than the flight cost benefit for the individual forager. We suggest that the ability to evaporate nectar outside the nest has a strong evolutionary benefit and shapes decision making during foraging on both individual and colony levels.

## Methods

### Sampling of nectar and honey bee crop contents

Honey bees were studied in macadamia orchards in the Barberton area, Mpumulanga Province (latitude: − 25.633146, longitude: 30.946245) during September 2020. Bees collected nectar from four different cultivars: hybrid cultivars 695 and A4 (nectar and forager crop contents sampled on 10 and 11 September 2020) and *Macadamia integrifolia* cultivars 814 and 816 (nectar and forager crop contents sampled on 28 and 29 September 2020). All cultivars were sampled during peak flowering. Racemes of the four cultivars are shown in Supplementary Fig. S1. Two groups of 40 hives each were placed on the farm for the pollination season (~ 600 m apart and in each case ~ 500 m from the edges of the trees). Positioning of the hives was specifically designed for this research project, with 40 hives placed near cultivars 695 and A4 (blocks adjacent to each other) and the other 40 near cultivars 814 and 816 (also adjacent to each other). The two groups of cultivars and their associated hives were separated by ~ 150 m of natural vegetation (lowveld region of the savanna biome).

For measurement of nectar concentrations, we selected 10 trees from each cultivar and pooled the nectar from 10 florets per raceme (total 30 racemes). The pendant racemes were picked between 13:00 and 14:00 and transported to a field laboratory for nectar removal. Nectar was collected using an Agilent syringe (10 µL) with a blunt point under a dissection microscope, and its concentration measured as % w/w sucrose equivalents with a pocket refractometer (Bellingham & Stanley Ltd, Tunbridge Wells, UK).

For each cultivar, nectar and forager crop contents were sampled on the same day. Returning foragers were also sampled on that day. Honey bees (30 per cultivar) were captured on flowers after they had been observed collecting nectar for 20–30 s and were low enough on the tree to catch. Sampling was carried out deep within the orchards and approximately 800 m from the hives, because the bees were very aggressive during macadamia flowering, especially near the hives. Bees were captured in plastic specimen sample bottles with screw tops (60 ml) and immediately put on ice. After cooling on ice for a maximum of 3 min, the crop was removed and its contents collected in glass haematocrit tubes (20 μL, Drummond Scientific). The volume of crop contents (µl) was obtained from the length of the fluid column and the concentration measured with the refractometer. We collected 30 bees per cultivar from 09:00 to 13:00, and racemes were picked for nectar measurements immediately afterwards.

In addition, we blocked the entrances of six hives on the same day in order to capture returning nectar foragers in a glass bottle (30 in total for the four cultivars after excluding pollen foragers, identified as those honey bees with pollen on their corbiculae, and guard bees with empty crops). Although returning bees were captured and sampled on the same days that foragers were sampled on each cultivar, it was not possible to tell which cultivar they had been foraging on. For this reason, the data for returning foragers have been pooled. Bees were placed on ice and the crops were removed at a distance of 800 m from the hives for measurements on crop contents as above.

Temperature and relative humidity were measured at ~ 10:00 on each sampling day with a hand-held Kestrel Weather Meter (Model Kestrel 3000, USA). The density of trees meant that little direct sunlight reached the racemes (Supplementary Fig. S2).

### Calculations: sugar and water in the honey bee crop (Table 1)

Sugar mass of the crop contents was calculated for each honey bee as the product of volume, concentration and density^14^. Total mass of the crop contents was obtained from the volume (Supplementary Table S1) and density, and the mass of water in the crop was the difference between total mass and sugar mass. For calculating the effect of evaporation, we assumed that the mass of sugar in the crop remained constant. Without evaporation, the total mass of crop contents was obtained by dividing the sugar mass by the mean nectar concentration (% w/w) of the cultivar on which the bees were foraging. Again, the mass of water in the crop was obtained by difference. The mass of water evaporated for each bee is then the difference in water mass between its measured crop contents and its crop contents if no evaporation occurred and the concentration remained the same as in the flowers. The percentage of water evaporated was derived from the mass of water removed from the crop contents and the mass of water in the crop assuming no evaporation.

### Calculations: energy savings due to evaporation

We calculated the energy expended during flight back to the hive, with or without evaporation of excess water, and also the energy saving due to elimination of water from fresh nectar. For the energy expended during flight we used the formula from Wolf et al.^12^ to calculate the oxygen consumption $VO_{2}$ in ml/h (Eq. 1).

1 $$VO_{2} = 0.514*m^{0.629}$$

where m is the total mass, i.e. the mass of the bee and the load she is carrying. The mass of the bee is taken as 84.9 mg (*A. m. scutellata*^41^).

E_va_, the energy (mJ) needed to return to the hive with evaporation from the crop contents, was calculated as:

2 $$E_{va} = 0.514*\left( {m_{b} + m_{c} } \right)^{0.629} *\frac{D}{V}*\frac{20.083}{{3600}}$$

where m_b_ is the mass of the bee, m_c_ is the recorded mass of the crop load (Table 1), D is the flight distance, V the flight speed and the last term is the energy per second. The flight distance D is 800 m and the flight speed V is 7.84 m/s^42^. The volume of oxygen (VO_2_) was converted to joules (J) using the conversion 1 ml O_2 _= 20.083 J.

If we assume evaporation of the crop contents does not occur:

3 $$E_{no} = 0.514*\left( {m_{b} + m_{n} } \right)^{0.629} *\frac{D}{V}*\frac{20.083}{{3600}}$$

where E_no_ = Energy needed for the return flight to the hive without evaporation of nectar (in mJ). Here m_n_ is the hypothetical crop load (Table 1) when keeping the amount of sugar constant but using the concentration of the flower nectar before evaporation by the bee.

The energy needed to evaporate the same quantity of excess water in the nest was calculated using the heat of vaporisation of water at 35 °C of 2.42 kJ/ml or 2.42 J/mg.

### Statistical analyses

Kruskal–Wallis ANOVA with multiple comparisons of mean ranks for all groups as a post hoc test. Data are shown as means and 95% confidence intervals. Groups were compared pairwise and non-overlapping confidence intervals were judged as significantly different. If, however, a pairwise comparison had overlapping confidence intervals, the confidence intervals for the differences of the two means were calculated to identify significant and non-significant differences between the two groups.

## Supplementary Information

Supplementary Information.

## Data Availability

Data are included in the supplementary material.
