# Supplementary material for: Honey bees save energy in honey processing by dehydrating nectar before returning to the nest
Source: Sci Rep. 2022 Sep 28;12:16224. doi: 10.1038/s41598-022-20626-5 (PMC9519551; doi:10.1038/s41598-022-20626-5)
Supplement: Supplementary file 1 — Supplementary Information. [file 41598_2022_20626_MOESM1_ESM.docx]

**Supplementary material**

**Honey bees save energy in honey processing by dehydrating nectar before returning to the nest**

Susan W. Nicolson, Hannelie Human and Christian W.W. Pirk

Department of Zoology and Entomology, University of Pretoria, Pretoria 0002, South Africa


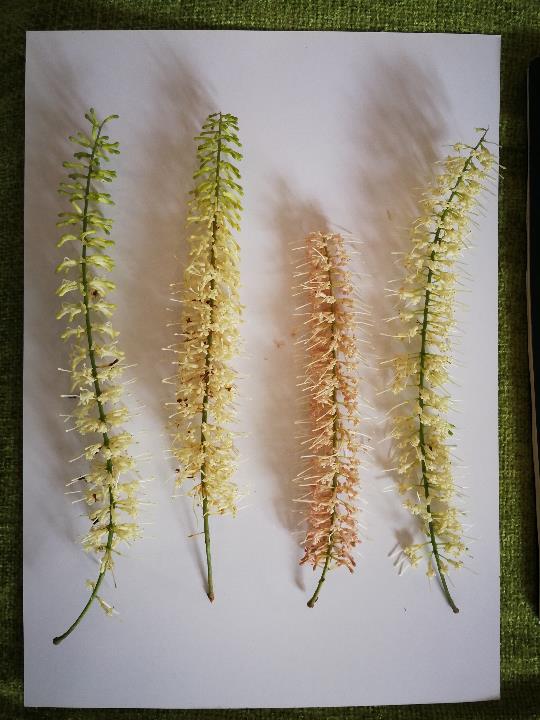
3

**Fig. S1** Racemes of the four *Macadamia* cultivars (left to right: 816, 814, 695, A4). For these cultivars, the average length of racemes ranged from 21 to 26 cm and the average number of florets ranged from 247 to 305 per raceme. Data were obtained from at least 25 racemes per cultivar.


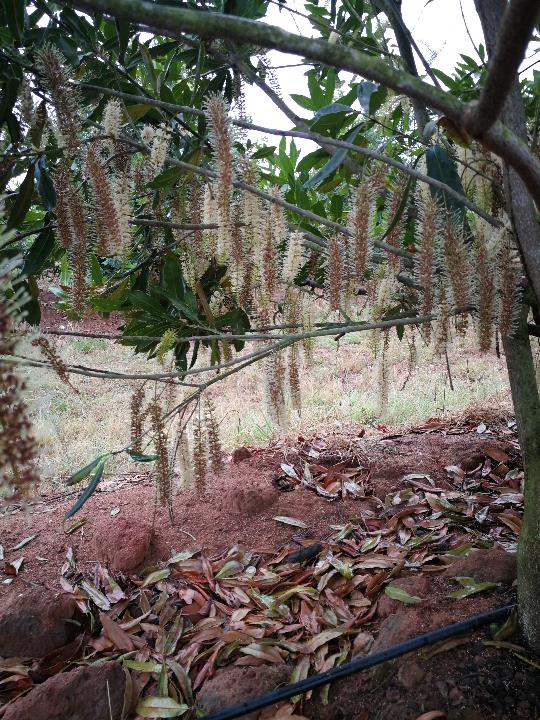


**Fig. S2.** Flowering *Macadamia* trees showing pendant racemes and shaded environment.

**Table S1.** Nectar and honey bee crop data. Nectar concentrations (% w/w) for the four *Macadamia* cultivars; volumes (µl) and concentrations (% w/w) of the crop contents of bees foraging on the four cultivars and captured on return to the hive.

| Cultivar | 695 |  |  | A4 |  |  | 814 |  |  | 816 |  |  | Returning bees |  |
| --- | --- | --- | --- | --- | --- | --- | --- | --- | --- | --- | --- | --- | --- | --- |
|  |  |  |  |  |  |  |  |  |  |  |  |  |  |  |
| RH & temp | 43.4% 26.7°C |  |  | 48.4% 30.4°C |  |  | 32.5% 26.6°C |  |  | 31.6%, 31.0°C |  |  | 48.4% 30.4°C |  |
|  | nectar | crop | crop | nectar | crop | crop | nectar | crop | crop | nectar | crop | crop | crop | crop |
|  | conc | volume | conc | conc | volume | conc | conc | volume | conc | conc | volume | conc | volume | conc |
|  |  |  |  |  |  |  |  |  |  |  |  |  |  |  |
|  | 19 | 18 | 41 | 25 | 18 | 42 | 27 | 15 | 61 | 30 | 17 | 46 | 24 | 66 |
|  | 20 | 12 | 42 | 28 | 15 | 58 | 26 | 17 | 63 | 33 | 12 | 54 | 20 | 64 |
|  | 22 | 10 | 37 | 25 | 26 | 33 | 26 | 24 | 63 | 33 | 25 | 34 | 18 | 60 |
|  | 23 | 18 | 37 | 20 | 20 | 36 | 28 | 15 | 58 | 31 | 20 | 50 | 18 | 67 |
|  | 24 | 20 | 38 | 20 | 19 | 38 | 27 | 18 | 62 | 27 | 15 | 38 | 22 | 55 |
|  | 22 | 12 | 34 | 22 | 26 | 35 | 26 | 20 | 61 | 28 | 14 | 45 | 17 | 57 |
|  | 23 | 16 | 41 | 22 | 20 | 45 | 28 | 22 | 63 | 27 | 25 | 58 | 15 | 63 |
|  | 21 | 21 | 32 | 24 | 16 | 45 | 30 | 17 | 58 | 28 | 12 | 58 | 24 | 65 |
|  | 20 | 24 | 31 | 20 | 18 | 38 | 29 | 20 | 64 | 30 | 17 | 56 | 17 | 70 |
|  | 20 | 19 | 32 | 22 | 20 | 40 | 30 | 22 | 64 | 26 | 20 | 56 | 15 | 67 |
|  | 19 | 19 | 35 | 20 | 24 | 42 | 31 | 24 | 62 | 31 | 17 | 59 | 25 | 65 |
|  | 20 | 11 | 37 | 21 | 20 | 40 | 31 | 20 | 63 | 27 | 24 | 58 | 20 | 67 |
|  | 20 | 18 | 36 | 21 | 16 | 34 | 28 | 19 | 60 | 27 | 12 | 60 | 21 | 63 |
|  | 19 | 12 | 36 | 21 | 20 | 36 | 30 | 20 | 66 | 35 | 17 | 57 | 15 | 62 |
|  | 21 | 10 | 40 | 21 | 22 | 36 | 30 | 17 | 60 | 30 | 25 | 61 | 20 | 67 |
|  | 19 | 23 | 43 | 22 | 16 | 37 | 30 | 15 | 65 | 28 | 22 | 63 | 24 | 60 |
|  | 23 | 21 | 41 | 20 | 17 | 34 | 30 | 10 | 60 | 26 | 25 | 63 | 19 | 67 |
| Table S1 continuous |  |  |  |  |  |  |  |  |  |  |  |  |  |  |
| Cultivar | 695 |  |  | A4 |  |  | 814 |  |  | 816 |  |  | Returning bees |  |
|  | nectar | crop | crop | nectar | crop | crop | nectar | crop | crop | nectar | crop | crop | crop | crop |
|  | conc | volume | conc | conc | volume | conc | conc | volume | conc | conc | volume | conc | volume | conc |
|  |  |  |  |  |  |  |  |  |  |  |  |  |  |  |
|  | 18 | 16 | 38 | 20 | 16 | 34 | 31 | 15 | 67 | 26 | 19 | 60 | 18 | 58 |
|  | 21 | 19 | 40 | 20 | 18 | 37 | 28 | 17 | 63 | 31 | 15 | 64 | 20 | 66 |
|  | 18 | 20 | 33 | 19 | 12 | 39 | 30 | 24 | 62 | 29 | 18 | 62 | 25 | 59 |
|  | 24 | 20 | 43 | 19 | 22 | 44 | 29 | 17 | 64 | 27 | 10 | 63 | 15 | 64 |
|  | 25 | 23 | 39 | 20 | 20 | 45 | 30 | 17 | 62 | 25 | 21 | 62 | 18 | 63 |
|  | 27 | 24 | 39 | 18 | 28 | 45 | 31 | 12 | 61 | 26 | 24 | 65 | 17 | 65 |
|  | 26 | 20 | 38 | 20 | 19 | 42 | 28 | 22 | 63 | 27 | 18 | 63 | 21 | 64 |
|  | 28 | 12 | 37 | 21 | 18 | 39 | 27 | 17 | 62 | 27 | 20 | 60 | 24 | 68 |
|  | 25 | 18 | 42 | 22 | 18 | 37 | 28 | 10 | 58 | 25 | 15 | 58 | 18 | 64 |
|  | 26 | 12 | 37 | 24 | 24 | 39 | 30 | 22 | 60 | 25 | 17 | 56 | 12 | 61 |
|  | 22 | 17 | 35 | 21 | 26 | 44 | 30 | 10 | 62 | 24 | 20 | 60 | 15 | 71 |
|  | 22 | 16 | 33 | 22 | 20 | 40 | 28 | 20 | 63 | 28 | 22 | 60 | 20 | 68 |
|  | 22 | 12 | 35 | 22 | 18 | 42 | 31 | 24 | 62 | 29 | 24 | 63 | 21 | 72 |
|  |  |  |  |  |  |  |  |  |  |  |  |  |  |  |
| Mean | 21.97 | 17.10 | 37.4 | 21.4 | 19.73 | 39.87 | 28.93 | 18.07 | 62.07 | 28.2 | 18.73 | 57.07 | 19.27 | 64.27 |

**Table S2.** Results of the Kruskal-Wallis ANOVA. In the case of an significant effect, right below are the pairwise multiple comparisons of mean ranks for all groups. For each multiple comparison the z-values are given above the grey diagonal and the corresponding p-values below it. Significant comparisons are highlighted in bold.

|  | Cultivar 695 | Cultivar A4 | Cultivar 814 | Cultivar 816 | Returning Bees |
| --- | --- | --- | --- | --- | --- |
| Nectar concentration between cultivars | | | Kruskal-Wallis test: H (3, N= 120) =81.84 p < 0.0001 | | |
| Cultivar 695 |  | 0.4705 | **6.5899** | **5.5837** |  |
| Cultivar A4 | n.s. |  | **7.0646** | **6.059** |  |
| Cultivar 814 | 0.0000001 | 0.0000001 |  | 1.005 |  |
| Cultivar 816 | 0.0000001 | 0.0000001 | n.s. |  |  |
| Crop volume between groups | | | Kruskal-Wallis test: H (3, N= 120) =5.38 p > 0.25 | | |
| Crop concentration among groups | | | Kruskal-Wallis test: H (4, N= 150) =110.57 p < 0.0001 | | |
| Cultivar 695 |  | 0.1322 | **6.6978** | **4.6906** | **8.0543** |
| Cultivar A4 | n.s. |  | **6.5656** | **4.5583** | **7.9221** |
| Cultivar 814 | 0.0000001 | 0.0000001 |  | 2.0072 | 1.3565 |
| Cultivar 816 | 0.000027 | 0.000052 | n.s. |  | **3.3638** |
| Returning Bees | 0.0000001 | 0.0000001 | n.s. | 0.007688 |  |
| Crop mass (mg) among groups | | | Kruskal-Wallis test: H (4, N= 150) =14.37 p < 0.0063 | | |
| Cultivar 695 |  | 1.8572 | 2.3847 | 2.5510 | **3.6594** |
| Cultivar A4 | n.s. |  | 0.5274 | 0.6939 | 1.8022 |
| Cultivar 814 | n.s. | n.s. |  | 0.1664 | 1.2747 |
| Cultivar 816 | n.s. | n.s. | n.s. |  | 1.1084 |
| Returning Bees | 0.0025 | n.s. | n.s. | n.s. |  |
| Sugar mass (mg) among groups | | | Kruskal-Wallis test: H (4, N= 150) =79.631 p < 0.0001 | | |
| Cultivar 695 |  | 1.257 | **5.9623** | **5.1541** | **7.3352** |
| Cultivar A4 | n.s. |  | **4.7054** | **3.8971** | **6.0782** |
| Cultivar 814 | **0.0000001** | **0.0000001** |  | 0.8082 | 1.372 |
| Cultivar 816 | **0.000003** | **0.0000973** | n.s. |  | 2.1811 |
| Returning Bees | **0.0000001** | **0.0000001** | n.s. | n.s. |  |
| Water mass (mg) among groups | | | Kruskal-Wallis test: H (4, N= 150) =57.19 p < 0.0001 | | |
| Cultivar 695 |  | 1.9612 | **4.1319** | 2.4827 | **4.1051** |
| Cultivar A4 | n.s. |  | **6.0931** | **4.4439** | **6.0663** |
| Cultivar 814 | **0.00036** | **0.0000001** |  | 1.6492 | 0.0267 |
| Cultivar 816 | n.s. | **0.000088** | n.s. |  | 1.6224 |
| Returning Bees | **0.000404** | **0.0000001** | n.s. | n.s. |  |
| Crop mass (mg) without evaporation among groups | | | Kruskal-Wallis test: H (4, N= 150) =57.08 p < 0.0001 | | |
| Cultivar 695 |  | 1.8721 | **3.9967** | **3.3905** | **7.1881** |
| Cultivar A4 | n.s. |  | 2.1246 | 1.5185 | **5.3161** |
| Cultivar 814 | **0.000642** | n.s. |  | 0.6062 | **3.1914** |
| Cultivar 816 | **0.007** | n.s. | n.s. |  | **3.7976** |
| Returning Bees | **0.0000001** | **0.0000001** | **0.01415** | **0.001461** |  |
| Water mass (mg) without evaporation among groups | | | Kruskal-Wallis test: H (4, N= 150) =50.94 p < 0.0001 | | |
| Cultivar 695 |  | 2.0533 | **2.9923** | 2.4901 | **6.9207** |
| Cultivar A4 | n.s. |  | 0.939 | 0.4368 | **4.8674** |
| Cultivar 814 | **0.028** | n.s. |  | 0.5022 | **3.9284** |
| Cultivar 816 | n.s. | n.s. | n.s. |  | **4.4306** |
| Returning Bees | **0.0000001** | **0.0000011** | **0.000855** | **0.000094** |  |
| Evaporated Water mass (mg) among groups | | | Kruskal-Wallis test: H (4, N= 150) =76.76 p < 0.0001 | | |
| Cultivar 695 |  | 1.673 | **4.5702** | **3.5778** | **8.1569** |
| Cultivar A4 | n.s. |  | **0.939** | 1.9048 | **6.4839** |
| Cultivar 814 | **0.000049** | **0.037645** |  | 0.9925 | **3.5866** |
| Cultivar 816 | **0.003466** | n.s. | n.s. |  | **4.5791** |
| Returning Bees | **0.0000001** | **0.0000011** | **0.00335** | **0.000047** |  |
